# Transcription of the Envelope Protein by 1-L Protein–RNA Recognition Code Leads to Genes/Proteins That Are Relevant to the SARS-CoV-2 Life Cycle and Pathogenesis

**DOI:** 10.3390/cimb44020055

**Published:** 2022-02-06

**Authors:** Jozef Nahalka

**Affiliations:** 1 Centre for Glycomics, Institute of Chemistry, Slovak Academy of Sciences, Dubravska Cesta 9, SK-84538 Bratislava, Slovakia; nahalka@savba.sk; 2 Centre of Excellence for White-Green Biotechnology, Institute of Chemistry, Slovak Academy of Sciences, Trieda Andreja Hlinku 2, SK-94976 Nitra, Slovakia

**Keywords:** protein–RNA recognition, bioinformatics method, COVID-19, SARS-CoV-2, identified genes, envelope protein

## Abstract

The theoretical protein–RNA recognition code was used in this study to research the compatibility of the SARS-CoV-2 envelope protein (E) with mRNAs in the human transcriptome. According to a review of the literature, the spectrum of identified genes showed that the virus post-transcriptionally promotes or represses the genes involved in the SARS-CoV-2 life cycle. The identified genes/proteins are also involved in adaptive immunity, in the function of the cilia and wound healing (EMT and MET) in the pulmonary epithelial tissue, in Alzheimer’s and Parkinson’s disease and in type 2 diabetes. For example, the E-protein promotes BHLHE40, which switches off the IL-10 inflammatory “brake” and inhibits antiviral T_H_αβ cells. In the viral cycle, E supports the COPII-SCAP-SREBP-HSP90α transport complex by the lowering of cholesterol in the ER and by the repression of insulin signaling, which explains the positive effect of HSP90 inhibitors in COVID-19 (geldanamycin), and E also supports importin α/β-mediated transport to the nucleus, which explains the positive effect of ivermectin, a blocker of importins α/β. In summary, transcription of the envelope protein by the 1-L protein–RNA recognition code leads to genes/proteins that are relevant to the SARS-CoV-2 life cycle and pathogenesis.

## 1. Introduction

**Figure 1:**
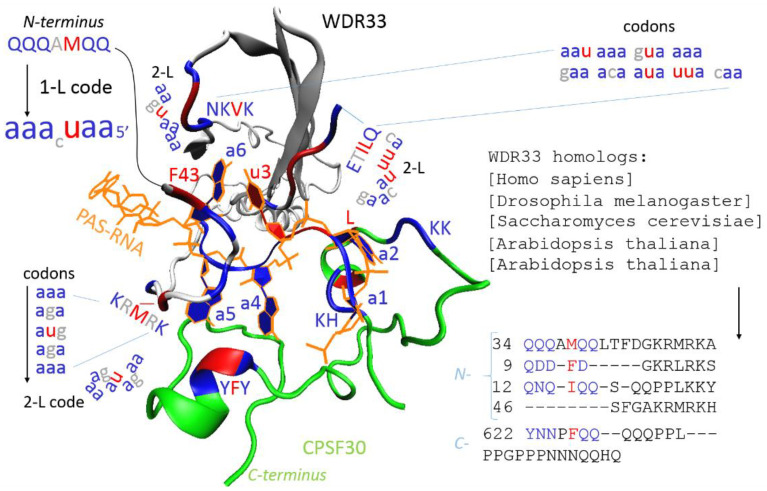
The principle of the proposed protein–RNA recognition code [9] is depicted in the interactions between the 5′AAUAAA hexamer and CPSF30 and WDR33 [11]. One-letter code—second nucleotide in the codons; two-letter code—first two nucleotides in the codons. Amino acids are in capitals, and nucleotides are in small letters. The compatibility to adenine is in blue, and the compatibility to uridine is in red. The PAS hexamer, poly(A) signal, is organized into three pairs (a1–a2, a4–a5 and u3–a6). In WDR33, the nucleotide sequence readout is performed by the KRMRK, NKVK and ETILQ sequences (2-L) and by the QQQAMQQ sequence (1-L). To read the 5′AAUAAA signal by 1-L, humans and plants use a “c” spacer (A or P), but *Drosophila* and *Saccharomyces* do not.

Whether the molecular RNA world existed or not, RNA represents the central molecule “software” (pre-mRNA) and “hardware” (e.g., ribosome), from the primary proto-cells to today’s eukaryotic cells. Proteins that bind RNA, RNA-binding proteins (RBPs), participate in the formation of ribonucleoprotein complexes (RNPs), and RNPs orchestrate mRNA maturation and protein translation processes. Besides participating in large RNP machines, spliceosomes and ribosomes, various RBPs can recognize hundreds of transcripts and form extensive regulatory networks that help to maintain cell homeostasis [1]. RBPs have real “housekeeping” functions and are more evolutionarily conserved in comparison with the transcription factors [1]. Not surprisingly, mutations or changes in their expression tend to cause various tissue-specific defects and diseases. In light of this, the functions and prognostic values of RBPs have been confirmed recently in various cancers [2,3,4,5] and neurodegenerative disorders [6]. In neurodegeneration, improperly processed RNA usually contains repeat sequences consisting of three to six nucleotides, which sequester RBPs specialized in the repeated sequence motifs [7] or initiates RAN translation (repeat-associated non-ATG), leading to toxic proteins [8]. For example, the (GGGGCC)n hexanucleotide repeat expansion in intron 1 of C9orf72 is a major cause of ALS (amyotrophic lateral sclerosis) and FTD (frontotemporal dementia) and the (GGGGCC)n sequester hnRNP H RBP [7] and generates insoluble polypeptides specific to FTD/ALS [8]. Studying these repeat expansions in various neurodegenerative disorders showed a nice agreement with the theoretically proposed protein–RNA recognition code [7,9]. The protein–RNA recognition code can also be depicted and explained on canonical pre-mRNA 3′-end processing [10]. In this process, the hexameric AAUAAA poly(A) signal (PAS) defines the 3′-end processing site, the cleavage and polyadenylation [10]. WDR33 and CPSF30 are two RBPs responsible for the readout of the PAS signal [11]. Both RBPs use intrinsically disordered regions, the *N*-terminus in WDR33 and *C*-terminus in CPSF30, which are semi-ordered upon binding of the PAS–RNA (Figure 1). Bonded PAS–RNA is recognized by amino acids that have compatible codons to the PAS hexamer sequence, U3 is read through amino acids that have U in the second position in codons and the adenine readout is performed by amino acids that have A in the second position in the codons (Figure 1); previously, this protein–RNA recognition mode was defined as the one-letter code (1-L) or the primary code [9,12]. The first two nucleotides in the codons were proposed as the two-letter code (2-L) [9] or the secondary code [12]. Structurally, CPSF30 recognizes A1–A2 and A4–A5 pairs, and WDR33 recognizes the U3–A6 Hoogsteen base pair, which is inserted between disordered NKVK and ETILQ sequences, and the A4–A5 pair, which is covered by a disordered segment near F43 (Figure 1, KRMRK). Usually, RBDs have a sequence of further distance but still close to the bound RNA, which can be transcribed by the 1-L code to the almost exact recognized RNA sequence; in this case, human WDR33 has the *N*-terminal QQQAMQQ sequence. These sequences are well-conserved (Figure 1, WDR33 homologs) and may be generally used for the identification of RBDs compatible with the recognizing RNA. In light of this, an RNA sequence can be transcribed to the various amino acid sequences that can be used for the protein BLAST search to identify the compatible RBDs [7]. In the opposite way, for example, a prion amino acid sequence has been transcribed to the imaginary RNA sequence, and a miRbase search revealed new microRNAs that have previously been identified as involved in prion diseases [7]. Similarly, three out of four SARS-CoV-2 structural proteins (S, M and N) were recently transcribed to the imaginary RNA sequences, and the imaginary sequences were applied to the BLASTn search in the human transcriptome in order to find genes/proteins whose expression could be modified by the expression of these viral proteins [13]. In this study, the envelope protein (E), the last SARS-CoV-2 structural protein, was processed by the same method. 

The E-protein is one of the most enigmatic proteins among the SARS-CoV-2 structural proteins and is associated with a multitude of immunopathological functions [14]. It was shown recently that E alone is able to cause acute respiratory distress syndrome (ARDS)-like damages in vitro and in vivo [15]. Physically, E forms a homopentameric cation channel (viroporin) [16], and the channel inhibitors reduced both the viral load and secretion of the inflammation cytokines [15]. The E-protein is a potential antiviral and vaccine target; this protein is highly conserved in the viral subtypes and highly expressed in infected cells [17]. However, only a small amount of E is incorporated into the viral structure, which indicates various functions [18]. E is the membrane protein that is targeted at ER–Golgi and ERGIC, respectively [18]. In light of protein–RNA interactions in the cell, cytosolic nonstructural/accessory proteins of SARS-CoV-2 would be much better and more appealing to involve in the viral modulation of the host cell protein–RNA interactome; however, according to the above described protein–RNA recognition code, E possesses exact compatibility with the UUUUUUUC polypyrimidine tract motif, which is one of the best-recognized sequence for HuR/ELAV1 RBP [19]. Human antigen R is an important post-transcriptional regulator of adipogenesis [20]; its knockdown is accompanied by a systemic glucose intolerance and insulin resistance, and this RBP is one of the first examples of an RBP directly antagonizing microRNA target regulation [21]. Theoretically, E is considered here as a RBP that recognizes and influences specific mRNAs or regulatory microRNAs. The external addition of purified E (the protein was stabilized in an aqueous solution by complexation with amphipols) undergoes surface-to-perinuclear retrograde trafficking, and the protein accumulates in the perinuclear space [22], which indicates that E could be involved in the regulation of mRNAs and microRNAs. 

Severe acute respiratory syndrome coronavirus 2 is known worldwide; it has caused the COVID-19 pandemic (Coronavirus disease 2019). The human coronavirus family includes four “established” coronaviruses (1960, HCoV-OC43 and HCoV-229E; 2004–2005, HCoV-NL63 and HCoV-HKU1), which cause mild respiratory disease and, after rhinoviruses, are a leading cause of common colds [23]. In light of this, the development of SARS-CoV-1/2 pathology was quite unexpected. As was mentioned above, the 1-L protein–RNA recognition code has been used previously for identification of the host cell genes influenced by SARS-CoV-2. The spectrum of the identified genes gives credence to both the proposed recognition code and the methodological pipeline used to identify the genes. For example, the persistence of viral RNA, pneumocyte syncytia and thrombosis are hallmarks of advanced COVID-19 pathology [24]; it is believed that the frequent generation of multinucleated cells (syncytia) results from activation of the SARS-CoV-2 spike protein (S) at the cell plasma membrane [25]. However, in addition to the S-initiated cell–cell fusion, it was found previously that the N protein post-transcriptionally promotes GCM1, which is a critical transcription factor promoting placental cell fusion (activates 1/2 syncytins and the fusogenic receptor MFSD2A) [13].

In summary, the basic protein–RNA interactome in a host–cell has an evolutionarily conserved “housekeeping” function, and “primitive” protein–RNA recognition is driven by intrinsically disordered regions that use the 2-L and 1-L protein–RNA recognition codes (Figure 1). Not surprisingly, viruses, such as the most primitive life forms, dysregulate this host cell protein–RNA interactome with their own proteins and RNA. Not surprisingly, small viral proteins must combine different functions and, among them, must be the “most primitive” 1-L protein–RNA recognition. For example, the small E-protein is partly the envelope protein of SARS-CoV-2, partly viroporin, and based on the 1-L code, E has exact compatibility with the polypyrimidine tract motifs, which are of the best recognition sequences for HuR/ELAV1 RBP. In this study, using the 1-L protein–RNA recognition code, a comparison of amino acid sequence E transcripts with the human transcriptome revealed virally dysregulated genes. These identified genes have been comprehensively investigated, and the functions and spectrum of the identified genes provide credibility for the theoretical recognition code and methodological approach used herein to identify dysregulated genes.

## 2. Methods

The rationale and motivation for the concept were explained in the introduction. In summary, the 1-L protein–RNA recognition code means that RNA-binding proteins (RBPs) use at least one amino acid sequence that is exactly compatible with the RNA nucleotide sequences according to the key one amino acid per nucleotide (1-L), and the nucleotide is defined by the type of nucleotide at the second position in the amino acid codon. For example, this can be illustrated by hexameric AAUAAA poly (A)-signal recognition by the *S. cerevisiae* WDR33 homolog (ScWDR33, Figure 1). The amino acid sequence *N*-Q12NQIQQ with the codon sequence *N*-CAA AAC CAA AUA CAA CAA reads PAS signal 5′-AAUAAA, so that, when the amino acid sequence ScWDR33 is transcribed into an imaginary nucleotide sequence using the 1-L code and is reversed, the AAUAAA PAS word can be identified in the sequence using nucleotide alignments. In other words, reversed *N*-QNQIQQ reads as 5′-AAUAAA. The method is very simple; each amino acid is transcribed to the second nucleotide of its codons, and the obtained imaginary RNA sequence is then applied to a BLASTn search of the human transcriptome to identify genes that can be post-transcriptionally regulated by the protein. 

S (Ser) has two possibilities for transcription, transcription to C (cytidine) or G (guanosine), and so two nucleotide sequences are obtained for each amino acid sequence, one with S–C transcription and the other with S–G transcription. As was mentioned above and shown in Figure 1, the 5′ RNA readout can be performed by the *N*-(AA)n-*C* amino acid sequence or by the reversed *C*-(AA)n-*N* amino acid sequence, so the transcription is done for two amino acid sequences, one for *N*-(AA)n-*C* and the second for *C*-(AA)n-*N*. In summary, four nucleotide sequences are obtained (please see the Supplementary Materials).

A BLASTn search in the human transcriptome was done as a standard nucleotide blast in the NCBI (https://blast.ncbi.nlm.nih.gov/Blast.cgi; accessed on 4 February 2022) for the four nucleotide sequences separately. “Genomic + transcript databases” and “Human genomic plus transcript”, “somewhat similar sequences” (blast algorithm), word size 7, max target sequences 500 and an expected threshold of 100 were used for the search. However, it is on the user of this method to choose word sizes 7 or higher/lower or change the other parameters, which are considered by the confidence level: “The perfect pairing—low number of hits; imperfect pairing—high number random hits and false positives”. 

Alignments with the gene transcript sequence are considered to be repressive (green in the figures), and alignments with the reverse complement sequences are considered to be promotive (yellow in the figures). 

## 3. Results

Throughout the whole study, it was considered that the viral structural protein E sequence is compatible with the mRNA of the protein host genes or with the host regulation microRNAs, and this was based on the protein–RNA recognition code. It is assumed that alignments with a complement sequence are the sequences that are responsible for post-transcriptional repression, because if the viral protein (or its degradation peptide) interferes with the host mRNA, then it represses the translation. It is assumed that alignments with reverse complement sequences are the sequences that are responsible for post-transcriptional promotion, because small RNAs or microRNAs with reverse complement sequences pair and bind to sites on the mRNAs in order to direct post-transcriptional repression; if the viral protein (or its degradation peptide) interferes with the host regulation RNA, then it promotes translation. Taken together, throughout the whole study, it was considered that the “primitive” primordial “protein–RNA recognition code” is currently operating in cells and is especially used in virus–host cell interactions in post-transcriptional regulation of the host cell mRNA.

**Figure 2:**
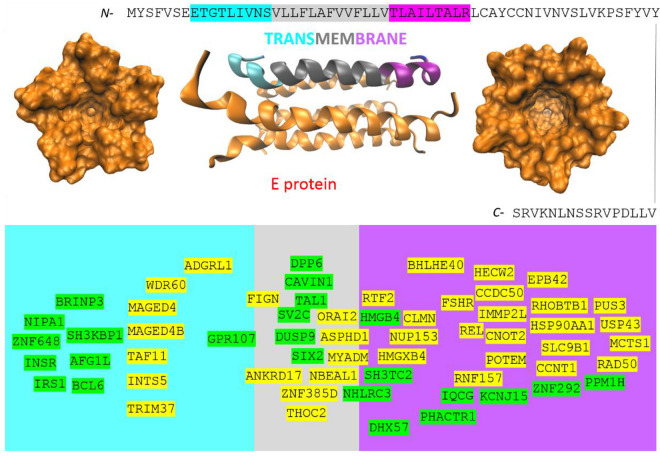
The SARS-CoV-2 envelope protein and the identified genes by 1-L transcription. Green highlights show the alignments with the complement sequence (post-transcriptionally repressed), and yellow highlights show the alignments with the reverse complement sequence (post-transcriptionally promoted). Alignments are compatible with the transmembrane domain (TMD); they can be divided into those compatible with the *N*-terminus of the TMD (highlighted in cyan), compatible with the *C*-terminus of the TMD (highlighted in magenta) and compatible with both ends of the TMD (highlighted in gray).

The envelope protein is a small integral protein critical for the life cycle of the *Coronaviridae* family. E is involved in the processes of virus assembly, budding and envelope formation, and mainly, it plays a key role in pathogenesis. E is a 75-residue homopentameric viroporin (Figure 2) that forms a cation-selective channel across the ERGIC membrane [16]. The ion-conducting function and pore formation is provided by the transmembrane domain (TMD) [16]. The whole E-protein consists of a short 7 amino acid disordered *N*-terminal region (NTR), 31 amino acid hydrophobic TMD and 37 amino acid long disordered hydrophilic carboxyl terminus (CTR) that resides cytoplasmically (Figure 2) [18]. Based on the presented theory, in the host or defense cells, E-protein post-transcriptionally promotes 37 genes (Figure 2, highlighted in yellow) and post-transcriptionally represses 24 genes (Figure 2, highlighted in green). Interestingly, no compatible sequence was localized outside of the TMD; all compatible sequences were fully/partially localized to the TMD (Supplementary Materials). The TMD can be divided into an *N*-terminal 8 amino acid sequence (Figure 2, highlighted in cyan), 13 amino acid core sequence (highlighted in gray) and *C*-terminal 8 amino acid sequence (highlighted in magenta). Based on the 1-L code, the TMD core sequence is transcribed as tttttCttttttt, the *N*-terminal TMD is transcribed as CGCTTTA-C/G and the *C*-terminal TMD is transcribed as CTCTTCCT. In light of this, the found genes can be distributed into three groups:

(1) CGCTTTA-C/G-tttttCttttttt-compatible (Figure 2, highlighted in cyan);
(2) tttttCtttttttCTCTTCCT-compatible (Figure 2, highlighted in magenta);
(3) compatible with both TMD ends (Figure 2, highlighted in gray).

## 4. Discussion

RBPs are typically thought of as proteins that bind RNA through one or multiple globular RNA-binding domains and change the fate or function of the bound RNAs. In the canonical view, protein–RNA recognition and binding by RBDs requires a stereospecific arrangement. However, Figure 1 shows that RNA can be recognized and bound by intrinsically disordered regions, uncovering unstructured polypeptides as regular partners in protein–RNA interactions. Disordered regions have emerged recently as frequent RNA interaction sites in vivo; half of the RBDs map to disordered regions [26]. These intrinsically disordered regions promote LLPS (liquid–liquid phase separation) via weak binding, and these interactions only require a few amino acids and do not require any stereospecific arrangement [27]. In light of this, intrinsically disordered NTR and CTR of E may be involved in the nucleotide sequence recognition by the 2-L code, and the TMD may be involved in nucleotide sequence recognition by the 1-L code (Figure 1), and the recognition can be done on the membrane periphery. For example, the transcription of the cytoplasmic *C*-terminal *N*-VLLDP sequence by 2-L is UGUUUUgaCC, where D/ga is the spacer. The transcription of TMD by the 1-L code gives specific uridine (T/t) and cytidine (C)-rich sequences of RNA (CGCTTTACtttttCtttttttCTCTTCCT), which could be specific targets for the host cell RBPs. As was mentioned before, HuR/ELAV1 RBP very well recognizes the tttttttC polypyrimidine tract motif [19]. In this view, the E-protein deregulates the host cell RBP–RNA interactome and may promote/repress about 61 genes. In the next section, the identified genes are comprehensively reviewed and discussed.

### 4.1. Altered Immune Homeostasis as a Consequence of SARS-CoV-2 Infection 

**Figure 3:**
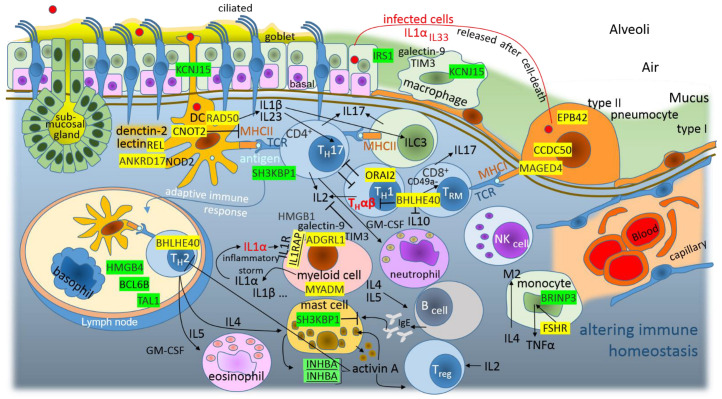
The graphic illustration of “Altered immune homeostasis as a consequence of SARS-CoV-2 infection”. Green highlights show the alignments with the complement sequence (post-transcriptionally repressed); yellow highlights show the alignments with the reverse complement sequence (post-transcriptionally promoted).

The human immune responses against pathogens and, in allergies/hypersensitivity, the various types of immune pathways are included, which have quite a wide spectrum of immune cells and are variable for each person. Despite the problematic categorization, it is useful to organize immune pathways logically into a summary diagram. In the traditional view, three basic lines are defined: type 1 immunity against intracellular pathogens (T_H_1 defense, viruses and intracellular bacteria, production of the IFN-γ cytokine and inflammation/autoimmunity); type 2 immunity against multicellular systems (T_H_2 defense, production of IL-4 and IL-5 cytokines and allergy/asthma) and type 3 immunity against extracellular pathogens (T_H_17 defense, bacteria and fungi, the production of IL-22 and IL-17 cytokines and inflammation/autoimmunity) [28]. Recently, this model was reclassified to the 4 × 2 + 2 immunological pathways [29]; however, to simplify things, the results are discussed here in reference to the above-mentioned basic three-line model. It was shown in a previous work that the S, M and N proteins purposely promote type 2 immunity against multicellular systems (allergy/asthma); however, it was also shown that the virus promotes a response to the IL-1 cytokine [13]. IL-1 activates mainly the T_H_17 defense, the type 3 immunity against extracellular pathogens [28,29]. It was shown that the N-protein has a compatible sequence for the post-transcriptional promotion of IL1RAP, the interleukin 1 receptor accessory protein, which is needed for IL1–IL1R1 signaling [13]. The infected cells release IL1α after their death; IL1α signals via IL1R on myeloid cells, promotes IL1–IL1R1 signaling and leads to further expression of IL1α and IL1β, and it can lead finally to self-perpetuating inflammation—an inflammatory storm (Figure 3) [30]. In addition, the E-protein promotes DNA damage sensor RAD50, which, in complex with the double-stranded DNA and the innate immune system adaptor CARD9, activates the transcription factor NF-κB and the production of IL-1β in dendritic cells (Figure 3) [31]. Dectin-2 lectin on dendritic cells selectively activates NF-κB subunit c-REL, which induces the expression of T_H_17-polarizing cytokines IL-1β and IL-23 [32]; interestingly, the E-protein promotes the exact c-REL type (Figure 3). Dendritic cells but, also, macrophages and mast cells use intracellular NLR-receptor NOD2, which senses Gram-positive peptidoglycan, and E-protein promotes ANKRD17, which is the positive regulator of NOD2-NF-κB inflammatory signaling [33,34]. ANKRD17 works also as a positive regulator of antiviral RLR-mediated immune signaling (RIGI-IRF3-IFNβ) [35,36]; however, it seems that the SARS-CoV-2 N-protein is able to capture ANKRD17 [37] and probably to shift its function to a Gram-positive bacterium. The E-protein also promotes CNOT2; the upregulation of CNOT2 may suppress and evade antiviral immunity [38]. In professional antigen-presenting cells, CNOT2 seems to mediate the repression of MHCII genes [39]. Taken together, the E-protein modulates intracellular NF-κB signaling to enhance the production of T_H_17-polarizing cytokines IL-1β and IL-23. NOD signaling and the IL-23–T_H_17 pathway (type 3) are known players in a number of chronic inflammatory diseases, for example, in inflammatory bowel disease (IBD) [34,40]. 

The E-protein promotes MYADM, myeloid-associated differentiation marker, which is upregulated when hematopoietic stem cells are induced to differentiate toward myeloid cells [41]. Acute myeloid leukemia cells (AMLs) express ADGRL1/LPHN1 (adhesion G protein-coupled receptor L1/latrophilin 1), which help to facilitate the secretion of galectin-9 (the immune suppressor that lacks a secretory domain) and TIM3 (T-cell immunoglobulin and mucin domain containing protein 3). This impairs the anti-AML leukocyte activities of cytotoxic lymphoid cells, and IL2 production is downregulated; additionally, the stress associated with these events leads AMLs to release HMGB1 (high-mobility group box 1), the protein that triggers the production of IL1β in healthy leukocytes [42]. The E-protein promotes ADGRL1/LPHN1; its promotion and the downregulation of IL-2 postpones the activation of T_reg_ “brake”, the host immune inhibitory cells that shift adaptive immunity to tolerable immunity [29]. In addition, the E-protein promotes the BHLHE40 transcription factor that switches off the IL-10, which is the inflammatory “brake” in all three types of adaptive immunity [43]. It was even shown recently that BHLHE40 is able to cooperate with the GM-CSF and IL-5 cytokines for the promotion of T_H_2 cell-mediated type 2 immunity [44]. Interestingly, BHLHE40 stimulates T_RM_ (tissue-resident memory CD8^+^ T) and T_IL_ (tumor-infiltrating lymphocyte) cell proliferation and functionality (Figure 3) [45]; however, the excessive accumulation of T_RM_ cells is not protective but, rather, drives inflammatory and fibrotic sequelae after primary respiratory viral infection [46]. Il-10-deficient mice spontaneously develop chronic inflammation, such as IBD [47].

The E-protein post-transcriptionally represses the SH3KBP1/CIN85 protein, which is important for the proper signaling of the T- and B-cell receptors (TCR and BCR) [48,49]. In T cells, SH3KBP1/CIN85 seems to be an inhibitor [48], but in B cells, it seems that SH3KBP1/CIN85 promotes BCR signaling and B-cell activation [49]. In mast cells, SH3KBP1/CIN85 overexpression accelerated the ligand-dependent endocytosis and degradation of the high-affinity receptor for IgE (FcϵRI), suggesting that CIN85 is an inhibitor of IgE–FcϵRI signaling [50]. Taken together, the E-protein represses the SH3KBP1/CIN85 protein, and this repression enhances TCR and FcϵR signaling but lowers BCR signaling and B-cell activation. 

The E-protein post-transcriptionally represses the BRINP3/FAM5C vascular inflammatory gene, and the low level of BRINP3 expression may be associated with ulcerative colitis [51] and peri-implantitis (inflammatory around Osseo-integrated dental implants) [52]. BRINP3/FAM5C is upregulated in response to inflammatory stimuli such as TNFα; interestingly, the E-protein promotes FSH receptor (FSHR), and FSH–FSHR signaling promotes a two to three-fold increase of TNFα transcription in monocytes [53]. The SiRNA knockdown of BRINP3/FAM5C inhibited the expression of endothelium-expressed ICAM1 (intercellular adhesion molecule-1) and VCAM1 (vascular cell adhesion molecule-1), resulting in reduced monocyte adhesion [54]. It seems that E-protein increases the production of the TNFα cytokine in monocytes and macrophages and activates other immune cells but decreases the monocyte and macrophage functionality, the adhesion to the epithelium/endothelium. In addition, the function of the electrically stimulated migration of the immune and endothelial cells to the infected sites (galvanotaxis/electrotaxis) is also abolished; the E-protein blocks K^+^ channel KCNJ15/KIR4.2, which is important for the sensing of weak electric fields and distributing of phosphatidylinositol 3,4,5-triphosphate to the leading edge in migrating cells (Figure 3) [55]. The E-protein represses IRS1, and as was mentioned above, E blocks TIM3-signalling by promotion of ADGRL1 and release of galectin-9 from myeloid cells. TIM3-signalling in macrophages and IRS1-PI3K-AKT-signalling in epithelial cells are both important for pulmonary epithelial barrier protection supplied by M2 macrophages [56]. In addition, E-protein promotes EPB42 (erythrocyte membrane protein band 4.2), the membrane protein that binds CD47 [57]. CD47 is the macrophage checkpoint that protect normal red cells from phagocytosis [58]. CD47 negatively regulates phagocytosis by acting as a “don’t-eat-me” signal [59], it seems that infected cell with SARS-CoV-2 could be masked by promotion of EPB42 and collecting of CD47.

E-protein represses HMGB4. Various HMGB1-antagonist are investigated to ameliorate HMGB1-mediated inflammation, relevant for COVID-19 treatment [60]. The chromatin-associated proteins known as high mobility group boxes (HMGBs) work extracellularly as DAMP (damage-associated molecular pattern) or alarmins which are released upon tissue damage and drive inflammatory responses. Based on HMGB1 prototype, it is believed that they bind to RAGE and TLR4 receptors and induces inflammations through activation of inflammasome and NF-κB signaling [60]. HMGB1-3 have two 80-amino acid HMG boxes and highly acidic *C*-terminal tail, nevertheless, HMGB4 lacks the acidic tail [61], which suggests different function. 

E-protein represses BCL6B inflammatory repressor. In mice, inactivation of Bcl6b promotes gastric cancer through amplification of the gastric inflammatory response [62]. Bcl6b b−/− mice had normal primary CD8^+^ T cell responses to influenza infection, but the response to reinfection had diminished cytotoxic T lymphocyte function, which was proportional to the lower frequency of specific CD8^+^ T cells, the absence of BCL6b seems to reduce the magnitude of the secondary response [63]. E-protein also represses TAL1/bHLHa17/SCL, T-cell acute lymphocytic leukemia protein 1, which is bHLH transcription factor important for development of all hematopoietic lineages [64], but mainly for the primitive erythropoiesis [65]. 

As was mentioned above, promoted BHLHE40 stimulates T_RM_ and T_IL_ cell proliferation, on the other hand, this factor inhibits the T_H_αβ cells, special T_H_1 cells, which provide the host immunity against viruses and their immune response is driven by IFNα/β and IL10 [29]. It was shown at previous work [13] that N protein represses DTX4, the inhibitor of type I interferon (IFN) signaling and antiviral immunity, on the other hand as is shown here, E-protein promotes BHLHE40 that “switch off” the IL10. In addition, E-protein promotes CCDC50, coiled-coil domain containing 50, known as Ymer, which is a negative regulator of type I IFN signaling. Upon RNA-virus infection, intracellular RLRs (retinoic acid-inducible gene I like receptors) sense cytosolic viral RNA and activates cascade for IFN-response [66]. IFN-response induces CCDC50 which recognizes K63-polyubiquitinated RLRs and targets them to the autophagosome for degradation. This autophagic cargo receptor works like a feedback control or a natural “decelerator” of the innate immunity [66]. It is evident that SARS-CoV-2 post-translationally promotes this protein to support CCDC50-mediated autophagy and deceleration of type I IFN antiviral signaling. 

Under inflammatory conditions, standard catalytic subunits of proteasome are replaced by interferon-inducible counterparts, and the immunoproteasome generated antigenic peptides are then loaded to the major histocompatibility complexes class I (MHCI) that are presented to CD8^+^ T cells [67], or the antigens are loaded to interferon-inducible MHCII that are presented to CD4^+^ T cells [68] (Figure 3). E-protein promotes MAGED4 and MAGED4B (melanoma antigen family members) which have tumor antigenic properties and elicit T-cell responses against tumor/tissue cells [69]. 

In summary, it seems that SARS-CoV-2 supports type 2 immunity against multicellular systems (T_H_2 defense) and type 3 immunity against extracellular pathogens (T_H_17 defense); as a consequence, there are both types of weakness type I immunity against intracellular pathogens (T_H_1 and T_H_αβ defense, intracellular bacteria and viruses). The adaptive immune responses seem to be virally reoriented to focus on tumor/host tissue and extracellular pathogens.

### 4.2. Altered Homeostasis in the Pulmonary Epithelial Tissue during the SARS-CoV-2 Infection 

**Figure 4:**
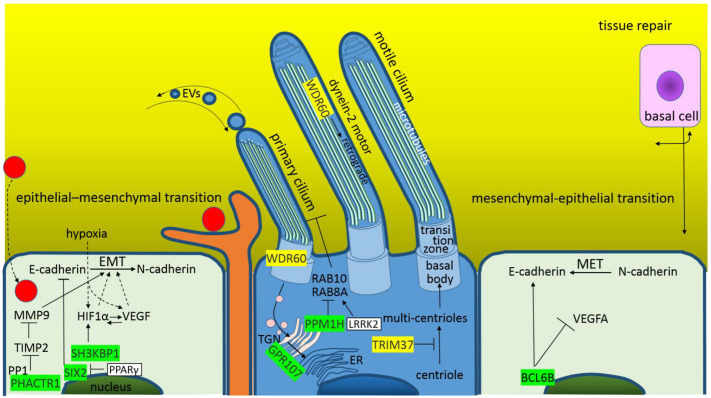
The graphical illustration of “Altered homeostasis in the pulmonary epithelial tissue during the SARS-CoV-2 infection”. Green highlights show the alignments with the complement sequence (post-transcriptionally repressed); yellow highlights show the alignments with the reverse complement sequence (post-transcriptionally promoted).

Epithelial cells line organs that are exposed to the outside world, the pulmonary epithelial tissue, continues from the trachea to the alveoli and lines the basement membrane. The airway epithelium is composed of four major cell types: “ciliated cells, goblet cells, secretory cells and basal cells” (Figure 3) [70]. During wound healing, epithelial cells use the epithelial–mesenchymal transition (EMT) and reverse mesenchymal–epithelial transition (MET) cellular programs (Figure 4). It is well-known that EMT and MET play crucial roles in the metastatic dissemination of carcinomas [71]. In a previous work [13], it was shown that both cellular programs are altered/inhibited during SARS-CoV-2 infection. It has also been shown that the M and N structural proteins may affect ciliogenesis and the function of the primary and motile cilium [13]. Similarly, E-protein alters the pulmonary epithelial tissue. E represses PHACTR1, SIX2 and SH3KBP1, which are involved in EMT. PHACTR1 is a phosphatase 1 and actin regulator; its downregulation enhances metalloproteinase inhibitors (e.g., TIMP2—the inhibition of migration-associated MMP9 and MMP2) [72,73]; SIX2 is a transcription factor that promotes lung cancer cell stemness and epigenetically regulates E-cadherin [74], and SH3KBP1/CIN85 is linked to hypoxia signaling; HIF1α degradation is promoted in its absence (Figure 4) [75]. In the case of MET, a metastasis suppressor BCL6B is repressed by E-protein; BCL6B upregulates cell adhesion gene E-cadherin and downregulates angiogenesis gene VEGFA (Figure 4) [76]. 

The E-protein promotes TRIM37 and represses PPM1H. The promoted TRIM37 is an E3 ubiquitin ligase identified as a negative regulator of ciliogenesis, which prevents the formation of centriolar protein assemblies [77] and centriole reduplication events [78]. The repressed PPM1H is an important phosphatase that dephosphorylates RAB proteins, and its knockdown suppresses primary cilia formation [79]. GPCR, which most closely resembles human GPR107, was identified in extracellular vesicles of *Chlamydomonas reinhardtii* (single-cell green alga) [80,81], and probably, cilia function as the “receiver and emitter” for the extracellular vesicles [82,83]. E-protein represses the mentioned GPR107 receptor, which finally localizes to the trans-Golgi network and is essential for retrograde transport [84]. On the other hand, E-protein supports the retrograde transport; it promotes WDR60, which heterodimerizes with WDR34 to the subcomplex, which is essential to form the dynein-2 complex [85]. Dynein-2 provides the trafficking of cargo along a microtubule in the retrograde direction, from the ciliary tip to the transition zone [86]. Dynein-2 is also involved in the transition zone assembly, maintenance and gating. WDR60 KO is associated with a reduced assembly of dynein-2, mislocalization of several ciliary membrane proteins and the accumulation of vesicles within the cilia [87]. It seems that SARS-CoV-2 E-protein supports the retrograde transport of vesicles through the cilia and from the cilia into the cell (WDR60), perhaps to obtain membranes, but blocks the vesicle retrograde transport to the Golgi–ER (GPR107).

### 4.3. Altered Neuronal Homeostasis as a Consequence of SARS-CoV-2 Infection 

A UK BioBank study showed recently that Alzheimer’s disease (AD) predicts the highest risk of SARS-CoV-2 infection and mortality among the elderly; in contrast, Parkinson’s disease (PD) patients were found to be at increased risk of infection, but not mortality, from COVID-19 [88]. The angiotensin-converting enzyme 2 (ACE2) receptors and proinflammatory markers such as IL-1 and galectin-9 (Figure 3) are common links with respect to COVID-19 and AD [89]. 

**Figure 5:**
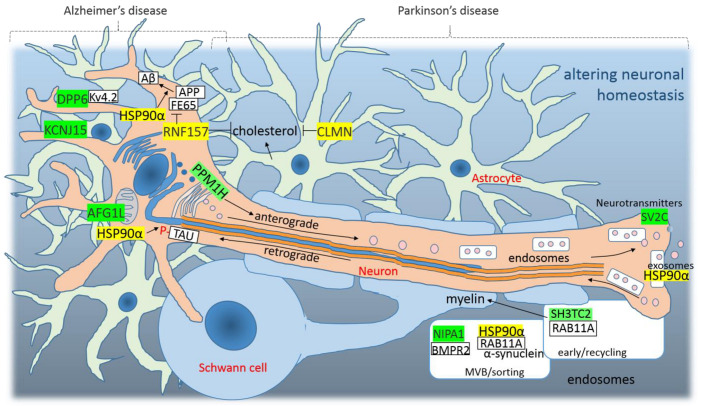
The graphical illustration of “Altered neuronal homeostasis as a consequence of SARS-CoV-2 infection”. Green highlights show the alignments with the complement sequence (post-transcriptionally repressed); yellow highlights show the alignments with the reverse complement sequence (post-transcriptionally promoted).

AD is related to the accumulation and deposition of β-amyloid (Aβ) peptides that are generated from the amyloid precursor protein (APP) by proteolytic processing with secretases [90]. The full-length membrane receptor APP shows cell-adhesive properties and promotes neuron viability and axonogenesis, and the APP intracellular domain (AICD) is responsible for the signaling [90]. APBB1/FE65 (amyloid beta precursor protein-binding, family B, member 1 or Fe65) mediates AICD-integrinβ interactions and AICD signaling/translocating to the nucleus [91]. Interestingly, E-protein promotes HSP90AA1/HSP90α and RNF157 (Figure 5). HSP90α facilitates Aβ processing and the aberrant aggregation [92] and RNF157 is the E3 ubiquitin ligase responsible for the degradation of APBB1/FE65 and dysfunction of the APP receptor (Figure 5) [93]. Interestingly, APP KO astrocytes have reduced cholesterol and have elevated levels of SREBP target gene transcripts [94]. In addition, HSP90α increases the accumulation of p-tau in neuronal tissue, especially in astrocytes, which is the second pathologic hallmarks of AD [95]. Human astrocytes are responsible for de novo cholesterol biosynthesis and regulation in the brain [94], a reduction in FE65 protein mimics the LRP (low-density lipoprotein receptor-related protein) knockout phenotype on APP processing [96]. In addition, E-protein promotes calmin (CLMN), its overexpression alone is sufficient to inhibit neuroblastoma (N2A) cell proliferation [97], and one GWA study strongly associated the CLMN gene with the reduction in the total cholesterol levels after statin treatment [98]. 

E-protein represses DPP6 and KCNJ15. Dipeptidyl peptidase like 6 is a membrane accessory subunit for the A-type K^+^ channel subunit Kv4.2 [99]. The loss of DPP6 function is involved in the dysfunction of neuronal excitability (hyperexcitability) [100], aging DPP6-KO mice have increased numbers of novel, abnormal presynaptic structures associated with several markers of AD [101], and the downregulation of DPP6 was associated with a reduced dendritic spine density, which is a phenotype observed in AD brains [102]. In addition, KCNJ15/KIR4.2 (potassium voltage-gated channel subfamily J member 15) gene variants were identified that showed suggestive associations with AD [103].

PD is defined by the selective loss of dopaminergic neurons. In PD, protein aggregation (pathogenic α-syn in Lewy bodies), mitochondrial damage and defects in endolysosomal trafficking are central factors [104]. The LRRK2 kinase, together with PPM1H phosphatase, control endolysosomal trafficking, the kination of RAB GTPases blocks GEF activators (guanine nucleotide exchange factors) and, then, the RABs stay in a GTP-bound state on the membrane, where they were phosphorylated; dephosphorylation of the RABs activates the transport (GEFs) [104]. Interestingly, E-protein represses PPM1H and SV2C. SV2C (synaptic vesicle glycoprotein 2C) belongs to a family of three proteins unique to secretory vesicles that undergo calcium-regulated exocytosis; SV2s regulate the ability of calcium to induce vesicle fusion in quiescent synapses and regulate the stability and trafficking of the calcium sensor protein synaptotagmin [105]. SV2C is the primary SV2 paralog in motor neurons [106] and dopaminergic neurons [105] and is required for normal levels of dopamine neurotransmission. SV2C interacts with synuclein, and the decreased expression of this regulator in striatal structures has been associated with PD [107]. Promoted HSP90AA1/HSP90α interacts with RAB11A and accelerates the fusion of multi-vesicular bodies (MVBs) with the plasma membrane and the subsequent release of exosomes [108,109]. In PD, the α-syn aggregates that block neurotransmitter release can be transferred to exosomes, which then mediate their propagation to the other cells [104]. E-protein represses SH3TC2 and NIPA1. SH3TC2/CMT4C is n effector of the endosomal recycling regulator RAB11; mutations that abrogate SH3TC2 and RAB11 interactions lead to the demyelination of Schwann cells in the peripheral nervous systems, and axon myelination is essential to impulse conduction in the vertebrate nervous system [110]. Repressed NIPA1/SPG6 is a magnesium transporter localized to endosomes and the plasma membrane. In hereditary spastic paraplegia, a neurodegenerative disease characterized by a motor neuron phenotype is thought that NIPA1/SPG6 inhibits BMP signaling by binding to the type II BMP receptor (BMPR2) and promoting its endocytic internalization and degradation [111]. In addition to defects in endolysosomal trafficking, E-protein represses AFG1L/LACE1, which is a mitochondrial integral membrane protein that preserves mitochondrial fidelity and cellular health. Its loss in *Caenorhabditis elegans* is associated with a reduced lifespan, reduced oxidative stress tolerance and with impaired mitochondrial proteostasis in the motor neurons [112].

### 4.4. SARS-CoV-2 Infection Alters Glucose Homeostasis to T2D 

There is strong evidence that type 2 diabetes (T2D) is associated with the worse outcome for COVID-19; T2D increases the mortality risk, and the properly controlled blood glucose correlates with the improved outcomes in infected patients [113]. T2D is a typical disease of advanced age, and therefore, it is not clearly known if COVID-19 is directly involved in T2D; however, COVID-19 might predispose infected individuals to hyperglycemia and T2D [114]. 

**Figure 6:**
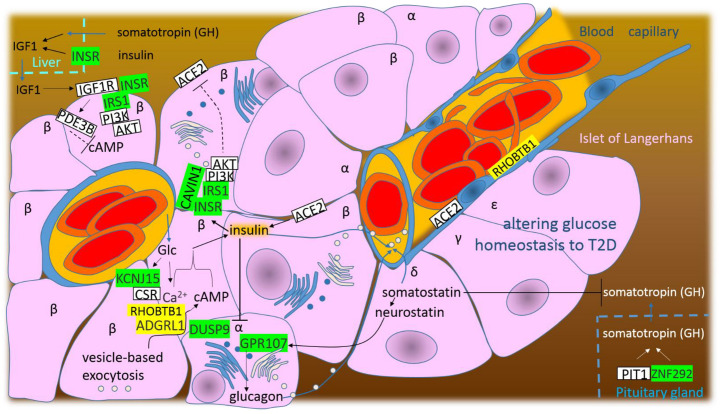
The graphical illustration of “SARS-CoV-2 infection alters glucose homeostasis to T2D”. Green highlights show the alignments with the complement sequence (post-transcriptionally repressed); yellow highlights show the alignments with the reverse complement sequence (post-transcriptionally promoted).

Based on this study, E-protein post-transcriptionally represses INSR, IRS1 and CAVIN1. The insulin physiological effects are mediated by binding to the insulin receptors (INSRs) on the plasma membrane (PM) of target cells; consequently, the induced conformational changes activate tyrosine kinase activity of the receptor, and then, metabolic and mitogenic signals are conducted [115]. In metabolic signaling, INSR activates/phosphorylates tyrosine residues of the insulin receptor substrate (IRS), which recruits and activates PI3K and AKT signaling effectors to propagate and amplify the signal (Figure 6) [115]. Although the six IRS isoforms have been identified, IRS1 and IRS2 mediate most of the metabolic effects [115]. CAVIN1 (the caveolae associated protein 1) is essential for caveola formation in vertebrate cells. In the presence of cholesterol and PI(4,5)P2, CAVIN1 homotrimers or CAVIN1/2 and CAVIN1/3 heterotrimers are incorporated to PM by caveolin (CAV1) oligomers, and then, they help to assembly the caveola [116]. The caveola is the membrane mechano-sensor, mechanical stress or insulin stimulation (phosphorylation of CAVIN1) induce of caveola disassembly and release the signaling cavins [116]. Insulin and glucagon signaling are both dramatically dampened in Cavin1−/− mice, the Cavin1 deficiency leads to lethal neonatal hypoglycemia [117].

E-protein promotes ADGRL1 and RHOBTB1. ADGRL1/LPHN1 (adhesion G protein-coupled receptor L1) belongs to a four member AGPCR-family known as latrophilins. Latrophilins are present in pancreatic islets and modulate insulin secretion, ADGRL1/LPHN1 signaling increases cAMP and the insulin exocytosis [118]. RHOBTB1 is Golgi localized RhoGTPase that serves as a substrate adaptor delivering phosphodiesterase 5 (PDE5) to the CUL3 E3 ring ubiquitin ligase complex for ubiquitination and degradation, hence, RHOBTB1 decreases cGMP and increases cAMP (Figure 6) [119]. The high glucose is metabolized in pancreatic β cells, and then, the increased ATP leads to depolarization and opening of calcium channels, and finally, the increased intracellular Ca^2+^ and cAMP induce secretion of insulin [118]. Interestingly, E-protein represses T2D–associated risk gene KCNJ15/KIR4.2, the potassium voltage-gated channel subfamily J member 15. KCNJ15 interacts with Ca^2+^-sensing receptor (CSR) and negatively regulates the insulin secretion. It seems that KCNJ15 works as a feedback control to protect against high insulin in the blood (hyperinsulinemia) [120].

In somatotropes, pituitary-specific positive transcription factor 1 (PIT1) activates the production of somatotropin (GH). E-protein represses transcriptional cofactor ZNF292/ZN15 that 5-fold increases GH-transcription above level of promotion by PIT1-alone [121]. In the liver, GH stimulates production of insulin-like growth factor 1 (IGF1) that is responsible for stimulating growth of all cell types [122], including pancreatic β cells, but in the case of β cells, the IGF1-IGF1R signaling activates phosphodiesterase PDE3B, which decreases cAMP and the insulin secretion [123]. 

E-protein represses DUSP9/MKP4 and GPR107. DUSP9/MKP4 is dual-specificity MAP kinase phosphatase that dephosphorylates/inactivates crucial mediators of stress-induced insulin resistance [124]. The overexpression of DUSP9/MKP4 clearly protects against stress-induced insulin resistance, in the mice liver, it decreases expression of gluconeogenic genes [124]. GPR107 (G protein-coupled receptor 107) is receptor adopted for neuronostatin (amidated 13-amino acid peptide) [125]. In the pancreas, neuronostatin is produced in δ-cells (from the somatostatin), and then, neuronostatin-GPR107 signaling initiates glucagon-production in α-cells (Figure 6) [80]. In light of the insulin secretion support described above, the virus appears to suppress glucagon-production, a potent inhibitor of insulin secretion, but promotes stress-induced insulin resistance and the expression of gluconeogenic genes (repressed DUSP9/MKP4).

The main entry receptor for SARS-CoV-2 is angiotensin-converting enzyme 2 (ACE2) [126] that produces angiotensin-(1–7) with anti-inflammatory, anti-oxidative, and neuroprotective effects, and actually, ACE2 is an opposite of ACE [114]. In the viral entry process, ACE2-internalization/consumption relatively increases ACE. Angiotensin II, the product of ACE, increases ROS, inflammation and reduces blood flow, and consequently, reduces the proliferation of the islet cells and the secretion of insulin [114,127]. It seems that the reduced islet cells and the reduced insulin are compensated by supporting of the insulin secretion, but on the other hand, the virus directly supports the insulin resistance (repressed INSR-IRS1 and CAVIN1 signaling). The ACE2/Ang-(1–7)/Mas axis can inhibit insulin resistance [128], it seems that promoted insulin resistance will promote ACE2 expression, and that is what the virus needs. Logically, T2D and other diabetic “predispositions” lead to an increased severity of COVID-19.

### 4.5. The Host Genes/Proteins Involved in the Life Cycle of SARS-CoV-2

Upon replication of vRNA in the cytoplasm and synthesis of structural proteins in the ER, coronaviruses perform RNA-encapsidation, capsid enveloping and the membrane budding into the lumen of ERGIC, the ER-Golgi intermediate compartment [23,129]. The assembly of viral envelope is coordinated by both M and E, but in the result, M predominantly and only a small portion of E is incorporated into the viral envelope of virions [18]. After the assembly, coronaviruses use Golgi-endosomal system for the transport and egress from the host cell [23]. 

**Figure 7:**
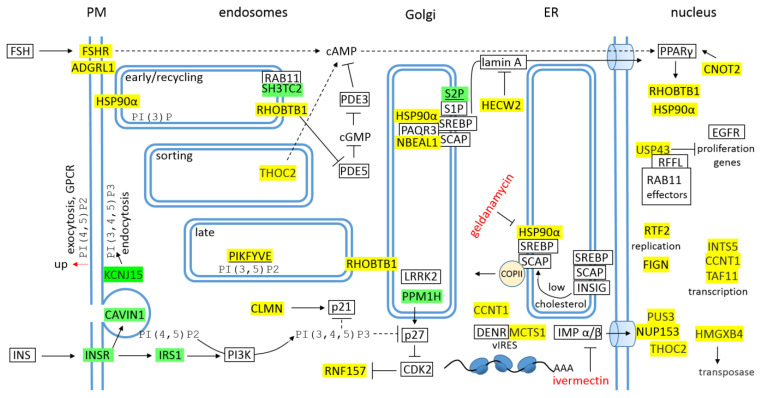
The graphical illustration/scheme of “The host genes/proteins involved in the life cycle of SARS-CoV-2”. Green highlights show the alignments with the complement sequence (post-transcriptionally repressed); yellow highlights show the alignments with the reverse complement sequence (post-transcriptionally promoted).

It was shown at the previous work that SARS-CoV-2 alters cholesterol homeostasis [13]. Interestingly, E-protein promotes NBEAL1 and HSP90α (Figure 7). NBEAL1 (neurobeachin like 1) is protein associated with the regulation of cholesterol homeostasis, it localizes into Golgi and interacts with the SREBP-cleavage activating protein (SCAP) and with the progestin and adipo-Q receptor 3 (PAQR3) [130], which both are involved in SREBP2-signaling [131]. In ER, SCAP senses cholesterol, and when the ER membrane cholesterol is “high” then it interacts with INSIG-protein (insulin-induced gene) and stay in the ER, when the ER-cholesterol is “low”, then it interacts with COPII vesicles and mediates transport of SREBP2 into the Golgi. In the Golgi, NBEAL1 and PAQR3 interacts with SCAP-SREBP2 complex and retains it in the Golgi membrane [131]. HSP90α binds to the SCAP–SREBP2 complex in the ER and facilitates its maturation and supports the transit of the complex to the Golgi, in the Golgi, HSP90α prevents premature degradation when the *N*-erminus of SREBP2 is cleaved by protease S1P. At last, S2P-protease releases the *N*-terminal domain, which then enters nucleus and activates transcription of promoters that contain SREs (sterol regulatory binding elements) [131]. It was shown at the previous work that the S-protein provides a sequence for post-transcriptional repression of S2P [13], and in addition, E-protein promotes E3 ubiquitin protein ligase HECW2/NEDL2, which ubiquitinates and mediates the proteasomal degradation of lamin (B/A) [132]. Lamin A is important for the recruitment and localization of active SREBP *N*-terminal domain to the nucleus [133]. Taken together, the virus supports COPII-SCAP-SREBP-HSP90α transport complex by the lowering of cholesterol (repressed S2P and promoted HECW2) and by the lowering of INSIG-proteins (repressed INSR-IRS1/CAVIN1 signaling, Figure 7). Actually, transcriptomic profiling of SARS-CoV-2 infected human cell lines identified HSP90 as target for COVID-19 therapy [134], and for example geldanamycin, the most studied and prominent HSP90 inhibitor, was proposed to treat COVID-19 (Figure 7) [134,135]. 

E-protein promotes FSHR and ADGRL1 receptors. FSHR, follicle stimulating hormone (FSH) receptor, belongs to the δ group of G protein–coupled receptor (GPCR) superfamily [136]. Interestingly, FSH-FSHR signaling activates PPARγ in surgically castrated male pigs [137] and promotes expression of Hsp90α in postmenopausal osteoporotic rats [138]. ADGRL1/LPHN1, as was mentioned above, belongs to a four-member adhesion GPCR-family known as latrophilins. LPHN1 is the most abundant isoform and its signaling increases cAMP and exocytosis [118]. In airway smooth muscle, KCNJ15/KIR4.2 is the major KIR channel [139] and its inhibition prevents phosphatidylinositol 3,4,5-triphosphate polarization in the plasmatic membrane [55]. Taken together, E-protein promotes receptors which increase cAMP and exocytosis, and on the other side, it represses INSR, IRS1 and KCNJ15 which increase PI(3,4,5)P3 and endocytosis (Figure 7). 

In addition to operating as a maturation and stabilization chaperone in COPII-SCAP-SREBP-HSP90α transport complex, HSP90α dimer (in open state) directly binds and deforms membranes and promotes the fusion of multi-vesicular bodies (MVBs) with PM and the subsequent release of exosomes [109]. As was already mentioned, E-protein promotes HSP90α and RHOBTB1. RHOBTB1 is Golgi localized RhoGTPase that serves as a substrate adaptor delivering phosphodiesterase 5 (PDE5) to the CUL3 E3 ring ubiquitin ligase complex for ubiquitination and degradation, hence, RHOBTB1 decreases cGMP and increases cAMP (Figure 7) [119]. Promoted nuclear THOC2, the largest subunit of TREX-complex, may be important for mRNA-export from nucleus, however, it positively influences cAMP-signaling [140] and was identified as a superior predictive exosome-derived molecular marker in hepatocellular carcinoma [141]. FSH-FSHR-cAMP signaling activates PPARγ in nucleus, and then, PPARγ closes the cycle, it promotes transcription of HSP90α and RHOBTB1 (Figure 7) [119,137,138]. It seems that both HSP90α and RHOBTB1 are important for the viral maturation and exocytosis. In addition, E-protein promotes CNOT2/NOT2 (CCR4-NOT transcription complex subunit 2) that activates PPARγ in 3T3-L1 preadipocytes (Figure 7) [142].

E-protein promotes ubiquitin specific peptidase 43 (USP43), which is a deubiquitylating enzyme associated with NuRD-complex (nucleosome remodeling deacetylase). USP43 coordinates the NuRD to repress genes which are involved in the cell proliferation [143]. USP43 also mediates degradation of effectors of the endosomal recycling regulators RAB11s [144]. These effectors elicit biological functions of RAB11s. The RAB11-subfamily comprises RAB11a, RAB11b and RAB11c/RAB25 members, which are localized to the recycling (RE), sorting (SE) and late (LE) endosomes. RAB11s are important in the trafficking of material from the TGN (trans-Golgi network) to endosomes, from SE to RE, and from RE to PM (anterograde transport) [145]. Interestingly, E-protein directly represses RAB11a effector SH3TC2/CMT4C, and both SH3TC2 and RAB11a are predominantly localized to REs. RAB11b has been shown to function in calcium induced exocytosis in neuronal and pancreatic β-cells [145]. RAB11s are also involved in endocytic trafficking [144], interestingly, RAB11a controls cholesterol transport from REs to the TGN. In Rab11a-depleted cells, the direct transport from REs to TGN was blocked and the cholesterol accumulated in the LE [146]. As was already mentioned, phosphorylation of RAB GTPases blocks GEFs-activators and RABs stay in the GTP bound state on the membrane where they have been phosphorylated, and then, dephosphorylation of RABs activates GEFs and the transport. In this way, RAB-specific LRRK2 kinase and PPM1H phosphatase control the endosomal transport (Figure 7) [104]. Interestingly, E-protein post-transcriptionally represses phosphatase PPM1H, and in this consequence, LRRK2 inhibits RABs and disrupts endocytic pathways [104].

In the nucleus, as was mentioned, promoted USP43 mediates inhibition of host cell proliferative genes, in addition, E-protein promotes RTF2 and FIGN. RTF2 (replication termination factor 2) was initially described in *Schizosaccharomyces pombe* as a mediator of site-specific replication termination, however recently, it was identified as a replisome component on elongating forks [147]. Under replication stress, RTF2 must be removed from stalled replication forks by DNA damage-inducible 1 (DDI1/2) protein, otherwise it causes massive ssDNA-formation (single-stranded DNA) and genome instability [147]. FIGN (fidgetin) is a microtubule severing enzyme translocated to nucleus, it preferentially targets highly-tyrosinated microtubules [148]. Fidgetin regulates cultured astrocyte migration [149]. Astrocytes exhibit Ca^2+^ mediated exocytosis of transmitters, and actually, astrocyte networks modulate breathing [150].

E protein promotes INTS5, CCNT1, and TAF11. INTS5 is a subunit in the integrator complex (INT), which is composed at least 14 subunits, and has role in RNA polymerase II (RNAPII) pause–release [151]. CCNT1 (cyclin T1) complexes with CDK9 kinase to form P-TEFb (positive transcription elongation factor b) that phosphorylates/activates paused RNAPII at most cellular genes [152]. P-TEFb interacts with other elongation factors to form super elongation complex (SEC), SEC interacts and cooperates with INT complex in the process of RNAPII pause–release [151]. On the other hand, promoted TAF11 (TATA-box binding protein associated factor 11) is inhibitory factor of the general transcription factor IID (TFIID). TFIID is a large multiprotein assembly that binds to all protein gene promoters and represents a key component of RNAPII-transcription initiation [153]. It seems that E-protein supports RNAPII pause–release but blocks transcription of host genes by promotion of inhibitory factor TAF11. Promoted CCNT1 also help to form separation liquid condensates [154], liquid–liquid phase separation (LLPS) generates high-density protein–RNA condensates, and seems that LLPS concentrates components of the SARS-CoV-2 replication machinery and form N-protein-vRNA condensates [155]. In additions, CCNT1 interacts with SARS-CoV-2 RNA [156].

E-protein promotes HMGXB4. HMGXB4/HMG2L1 (HMG-box containing 4) is transcriptional factor that 10- to 15-fold increases the transcription of transposase in sleeping beauty DNA-transposon sequence [157]. Additionally, to upregulation of transposase in DNA-transposon, in SARS-CoV-2 infected human cells, retro-transposon upregulation can be observed [158]. Transposons or the transposable elements (TEs) are genomic parasites that are found in all genomes. HMGXB4 also participates as the most abundant protein in the NuRF (nucleosome remodeling factor)-complex, NuRF is a candidate for the host-encoded factor required for TE-transposition and to assist viral life cycle [159]. It seems that the promotion of HMGXB4 supports human genome invasion with parasitic fragments and decrease the host-cell immunity.

E protein promotes NUP153, THCO2, and PUS3. NUP153 (nucleoporin 153) is nuclear basket nucleoporin which is required for the localization and anchoring of the other nuclear basket components (e.g., NUP50, TPR) to the nuclear pore complex [160]. In the absence of NUP153, importin α/β mediated transport to nucleus is strongly reduced [161]. Interestingly, ivermectin’s antiviral action on SARS-CoV-2 lays on the preventing importin α/β from binding to the viral proteins and blocking of their transport to the nucleus [162]. THOC2 (THO complex subunit 2) is the largest subunit of TREX-complex, it probably acts as a scaffold for the formation of the complex, minimally as the scaffold for its THO-subcomplex [163]. TREX-complex is transcription and export complex functionally linked to each mRNA processing step [163]. PUS3 (pseudouridine synthase 3) modifies U38/39 in both cytoplasmic and mitochondrial tRNAs [164]. Using PUS3Δ yeast mutants lacking Ψ38/39-modification, it was shown that Ψ38/39-modification is needed for efficient -1 frameshifting and this frameshifting is important in many viruses including Coronavirus [165]. In transcription of SARS-CoV-2 vRNA, -1 ribosome frameshift occurs immediately upstream of the ORF1a stop codon and the following translation of ORF1b yields in a large polypeptide (pp1ab), which is cleaved by viral proteases (nsp3 and nsp5) into 15 nsp proteins [166]. In addition, incorporation of Ψ attenuates cellular innate immune responses to transfected mRNAs and increases their translational capacity and stability [167]. It seems that SARS-CoV-2 uses U/Ψ-modification frequently, especially in E mRNA, no A,C,G-modification but only U-modification is observed [166].

E protein promotes MCTS1/MCT1. The malignant T cell-amplified sequence 1 forms a heterodimer with DENR (density-regulated protein) and serves as the translation factor which supports noncanonical translation initiation and ribosome recycling linked to cancer, viral infection, and neurological disorders [168]. During a viral infection and the activation of immune responses, standard eukaryotic initiation factor (eIF2) is under strict control but DENR-MCT1 complex can be used to translation via ribosome recycling at the viral IRES (internal ribosome entry site), for example in the HCV-IRES [169].

The anaphase promoting complex (APC) is a multi-subunit ubiquitin ligase that facilitates mitotic and G1 progression and is responsible for maintaining genomic stability [170]. E-protein promotes APC-CDH1 (coactivator) specific substrate RNF157. As was already mentioned (Figure 5), RNF157 is E3 ubiquitin ligase responsible for the degradation of APBB1/FE65 and the dysfunction of APP receptor [93]. APP is involved in the endocytosis of LDL receptor ligands and its cytoplasmic adaptor protein FE65 has been shown to link the *C*-terminal endocytosis motifs of APP and LDL receptors [94]. APP KO astrocytes have reduced cholesterol and elevated levels of SREBP [94]. In addition, E-protein promotes calmin (CLMN), one GWA-study strongly associated CLMN gene with the reduction in total cholesterol levels after statin treatment [98]. Contrary to oncogenic RNF157, overexpression of CLMN is sufficient to inhibit cell proliferation by upregulation of p21/CIP1 (Figure 7) [97].

## 5. Conclusions

This study completed a theoretical analysis of SARS-CoV-2 structural proteins by a protein–RNA recognition code. Compared to other structural proteins, the smallest E-protein is compatible with the largest group of human genes/proteins. The structural proteins S, M and N have previously been shown to support type 2 immunity against multicellular systems (T_H_2 defense) and reorient adaptive immunity to target host tissue (allergy and inflammation), but in addition to T_H_2 defense, the E-protein promotes type 3 immunity against extracellular pathogens (T_H_17 defense). Both of the elicited immunity types, type 2 and 3, actually repress type 1 immunity against intracellular pathogens (T_H_1 and T_H_αβ defense, intracellular bacteria and viruses). In addition, promoted BHLHE40 switches off the IL-10 inflammatory “brake” and stimulates the proliferation of (tumor) tissue-infiltrating lymphocytes and inhibits antiviral T_H_αβ cells, which are driven by IL-10 and IFNα/β.

The human airway epithelium is composed of four major cell types: “ciliated cells, goblet cells, secretory cells and basal cells”; during wound healing, these epithelial cells use the epithelial–mesenchymal transition (EMT) and reverse mesenchymal–epithelial transition (MET). Like other structural proteins of SARS-CoV-2, E inhibits both cellular programs involved in the wound healing and also negatively alters the epithelium cleaning and ciliogenesis. For example, E promotes TRIM37, which is the negative regulator of ciliogenesis.

Alzheimer’s disease (AD) predicts the highest risk of SARS-CoV-2 infection and mortality among the elderly; in contrast, Parkinson’s disease (PD) patients were found to be at increased risk of infection, but not mortality, from COVID-19 [88]. It was shown here that E dysregulates the genes associated with AD or PD. For example, promoted HSP90α facilitates APP processing to Aβ and the aberrant aggregation of Aβ (AD), increases the accumulation of p-tau in neuronal tissue (AD) and mediates the fusion of multivesicular bodies with the plasma membrane and the subsequent release of exosomes, the exosomes containing α-syn aggregates facilitate spreading the aggregates to other cells (PD).

There is strong evidence that type 2 diabetes (T2D) is associated with the worse outcome for COVID-19; T2D increases the mortality risk, and properly controlled blood glucose correlates with improved outcomes in infected patients [113]. This theoretical study showed that the virus promotes insulin secretion, insulin resistance and the expression of gluconeogenic genes. Hyperinsulinemia and hyperglycemia will probably induce the expression of the ACE2 regulator, and this is “what the virus consumes” for entry and propagation. Thus, which is the culprit for the worse outcome in patients with COVID-19 and diabetes, hyperglycemia or insulin? Maybe both; insulin treatment is associated with a significant increase in the death rate in patients with COVID-19 and T2D [171].

In light of the viral life cycle, the virus controls cholesterol entry, transport and synthesis. Insulin resistance and the lowering of INSIG proteins and the lowered cholesterol in ER support COPII-SCAP-SREBP-HSP90α transport, but the final step of the initiation of cholesterol synthesis, the release and transport of the SREBP-*N*-terminal domain to the nucleus, is blocked by the virus to provide the continuation of this transport. Interestingly, laminoma-mediated transport to the nucleus is blocked by the virus, with a focus on repressing SREBP-*N*-terminal domain transport into the nucleus, but importin α/β-mediated nuclear transport is promoted by the virus. Actually, geldanamycin (its derivatives) and ivermectin are known as the COVID-19 treatments [134,162]; geldanamycin is the inhibitor of HSP90α, and ivermectin prevents importin α/β from binding to the viral proteins. Transcriptomic profiling and analysis of the protein–RNA interactions of SARS-CoV-2-infected human cell lines identified HSP90 as a target for COVID-19 therapy [134,172], which gives credence to the theoretical recognition code and the methodological pipeline used here to identify the genes. 

## Data Availability

Not applicable.

## Supplementary Materials

The following are available online at https://www.mdpi.com/article/10.3390/cimb44020055/s1, Figure S1: Alignments of the identified genes by 1-L code *N*-AA-*C* and *C*-AA-*N* transcriptions.
