# Supplementary material for: Transcription of the Envelope Protein by 1-L Protein–RNA Recognition Code Leads to Genes/Proteins That Are Relevant to the SARS-CoV-2 Life Cycle and Pathogenesis
Source: Curr Issues Mol Biol. 2022 Feb 6;44(2):791–816. doi: 10.3390/cimb44020055 (PMC8928949; doi:10.3390/cimb44020055)
Supplement: Supplementary file 1 [file cimb-44-00055-s001.zip › cimb-1511583-supplementary.pdf]

Supplementary, *Current Issues in Molecular Biology*,

Transcription of the envelope protein by 1-L protein-RNA recognition code leads to genes/proteins that are relevant to the SARS-CoV-2 life cycle and pathogenesis

Jozef Nahalka

[1] *Institute of Chemistry, Centre for Glycomics, Slovak Academy of Sciences, Dubravska cesta 9, SK-84538 Bratislava, Slovak Republic;* [2] *Institute of Chemistry, Centre of excellence for white-green biotechnology, Slovak Academy of Sciences, Trieda Andreja Hlinku 2, SK-94976 Nitra, Slovak Republic*

**Correspondence: nahalka@savba.sk*

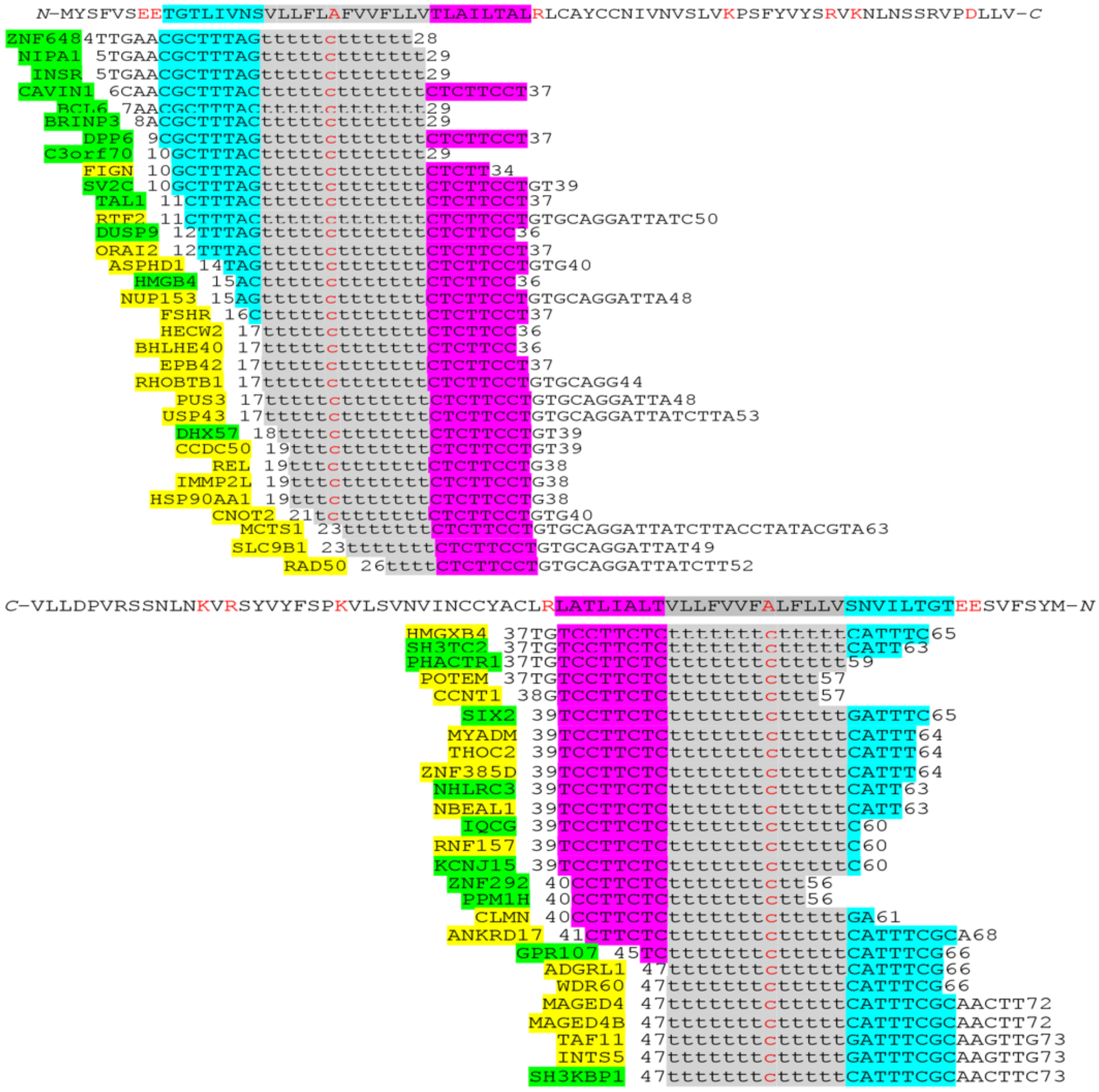

1 Supplementary Figure S1 Alignments of the identified genes by 1-L code *N*-AA-*C* and *C*-AA-*N* transcriptions.

2 Supplementary sequence information

>QHD43418.1 envelope protein [Severe acute respiratory syndrome coronavirus 2]
MYSFVSEETGTLIVNSVLLFLAFVVFLLVTLAILTALRLCAYCCNIVNVSLVKPSFYVYSRVKNLNSSRVPDLLV

E protein, blastn (Human G+T; somewhat similar sequences) for envelope protein and hsa Transcripts, "word size 7", "expect threshold 100" and "max target sequences 500"

Ec transcription, S (Ser) transcription to C (cytidine)
UACUUCAACGCUUUACUUUUUCUUUUUUUCUCUUCCUGUGCAGGAUUAUCUUACCUAUACGUAAUACCGUCAUUU

>NM_012232.5:3392-3423 Homo sapiens caveolae associated protein 1 (CAVIN1), mRNA

```
Query  6     CAACGCTTTACttttctttttttCTCTTCCT  37
             ||| ||||| |||||| ||||||| |||||||
Sbjct  3392  CAAAGCTTTGCTTTTTTTTTTTTTTCTTCCT  3423
```

>NM_020760.3:6916-6935 Homo sapiens HECT, C2 and WW domain containing E3 ubiquitin protein ligase 2 (HECW2), transcript variant 1, mRNA

```
Query  17    ttttctttttttCTCTTCC  36
             ||||||||||||||||||||
Sbjct  6935  TTTTCTTTTTTTCTCTTCC  6916
```

>NM_174908.3:953-973 Homo sapiens coiled-coil domain containing 50 (CCDC50), transcript variant 1, mRNA

```
Query  19   tttctttttttCTCTTCCTGT  39
            ||||| |||||||||||||||
Sbjct  973  TTTCTCTTTTTCTCTTCCTGT  953
```

>NM_001126340.2:9180-9205 Homo sapiens ORAI calcium release-activated calcium modulator 2 (ORAI2), transcript variant 1, mRNA

```
Query  12    TTTACttttctttttttCTCTTCCT  37
             ||| ||||| |||| |||||||||||
Sbjct  9205  TTTTCTTTTCCTTTCTTTCTCTTCCT  9180
```

>NM_000119.2:2531-2551 Homo sapiens erythrocyte membrane protein band 4.2 (EPB42), transcript variant 1, mRNA

```
Query  17    ttttctttttttCTCTTCCT  37
             ||||| |||||||||||||||
Sbjct  2551  TTTTTTTTTTTTCTCTTCCT  2531
```

>XM_005247694.4:470-492 PREDICTED: Homo sapiens BCL6 transcription repressor (BCL6), transcript variant X1, mRNA

```
Query  7    AACGCTTTACttttcttttttt  29
            |||||||||||||||| || ||
Sbjct  470  AACGCTTTACTTTTTCTCTTCTT  492
```

>NM_014836.4:2453-2480 Homo sapiens Rho related BTB domain containing 1 (RHOBTB1), transcript variant 1, mRNA

```
Query  17    ttttctttttttCTCTTCCTGTGCAGG  44
             ||||  ||||||||||||||| | ||||
Sbjct  2480  TTTTGTTTTTTTCTCTTCCTCTTCAGG  2453
```

```
>NM_002908.3:5051-5070 Homo sapiens REL proto-oncogene, NF-kB subunit
(REL), transcript variant 1, mRNA
Query  19    tttctttttttCTCTTCCTG  38
             ||||||||||| ||||||||
Sbjct  5070  TTTCTTTTTTTTTCTTCCTG  5051


>NM_001199303.1:3318-3337 Homo sapiens CCR4-NOT transcription complex
subunit 2 (CNOT2), transcript variant 1, mRNA
Query  21    tctttttttCTCTTCCTGTG  40
             ||||||||||||||||| ||
Sbjct  3337  TCTTTTTTTCTCTTCCTATG  3318

>NM_001025266.2:4674-4693 Homo sapiens chromosome 3 open reading frame 70
(C3orf70), mRNA
Query  10    GCTTTACttttctttttttt  29
             |||||||||||| |||||||
Sbjct  4674  GCTTTACTTTTTTTTTTTTT  4693

>XM_017012699.1:966-985 PREDICTED: Homo sapiens inner mitochondrial
membrane peptidase subunit 2 (IMMP2L), transcript variant X1, mRNA
Query  19   tttctttttttCTCTTCCTG  38
            ||| ||||||||||||||||
Sbjct  985  TTTTTTTTTTTCTCTTCCTG  966

>NM_001283035.1:486-525 Homo sapiens replication termination factor 2
(RTF2), transcript variant 1, mRNA
Query  11   CTTTACttttcttttttttCTCTTCCTGTGCAGGATTATC  50
            ||||| | |||| ||| | | |||||   |||||||||||
Sbjct  525  CTTTAGTGTTTCCTTTATCCCCTTCCCAGGCAGGATTATC  486

>XM_017004419.1:6399-6423 PREDICTED: Homo sapiens fidgetin, microtubule
severing factor (FIGN), transcript variant X2, mRNA
Query  10    GCTTTACttttcttttttttCTCTT  34
             |||||||||||||  |||| |||||
Sbjct  6423  GCTTTACTTTTTCCATTTTGCTCTT  6399

>NM_001017963.2:2392-2411 Homo sapiens heat shock protein 90 alpha family
class A member 1 (HSP90AA1), transcript variant 1, mRNA
Query  19    tttctttttttCTCTTCCTG  38
             ||| ||||||||||||||||
Sbjct  2411  TTTGTTTTTTTCTCTTCCTG  2392

>NM_031307.3:1831-1861 Homo sapiens pseudouridine synthase 3 (PUS3),
transcript variant 1, mRNA
Query  17    ttttcttttttttCTCTTCCTGTGCAGGATTA  48
             ||||| ||||||| | || || ||||||||||
Sbjct  1861  TTTTTTTTTTTTTTTTTTACT-TGCAGGATTA  1831

>NM_014060.2:4614-4655 Homo sapiens MCTS1, re-initiation and release factor
(MCTS1), transcript variant 1, mRNA
Query  23    tttttttCTCTTCCTGTGCAGGATTAT-CTTACCTATACGTA  63
             || || ||||||||| || || ||||| ||||   |||||||
Sbjct  4655  TTCTTATCTCTTCCTCTGAAGTATTATACTTAGAAATACGTA  4614

>NM_003670.2:3026-3045 Homo sapiens basic helix-loop-helix family member
e40 (BHLHE40), mRNA
```

```
Query  17    tttttctttttttCTCTTCC  36
             ||||| ||||||||||||||
Sbjct  3045  TTTTTTTTTTTTTCTCTTCC  3026
```

>XM_006714093.3:247-273 PREDICTED: Homo sapiens solute carrier family 9 member B1 (SLC9B1), transcript variant X1, mRNA

```
Query  23   tttttttCTCTTCCTGTGCAGGATTAT  49
            ||||  | ||||||||||||| |||||
Sbjct  273  TTTTAGTTTCTTCCTGTGCAGTATTAT  247
```

>NM_198963.2:4701-4722 Homo sapiens DExH-box helicase 57 (DHX57), transcript variant 1, mRNA

```
Query  18    ttttctttttttCTCTTCCTGT  39
             |||| ||||||||| |||||||
Sbjct  4701  TTTTTTTTTTTTCTTTTCCTGT  4722
```

>XM_011532733.2:5593-5614 PREDICTED: Homo sapiens follicle stimulating hormone receptor (FSHR), transcript variant X1, mRNA

```
Query  16    CtttttctttttttCTCTTCCT  37
             |||||||| || ||||||||||
Sbjct  5614  CTTTTTCTCTTATTCTCTTCCT  5593
```

>XM_005271160.4:1263-1289 PREDICTED: Homo sapiens TAL bHLH transcription factor 1, erythroid differentiation factor (TAL1), transcript variant X1, mRNA

```
Query  11    CTTTACtttttctttttttCTCTTCCT  37
             |||| |||||||| | | |||||||||
Sbjct  1263  CTTTTCTTTTTCTGTCTCTCTCTTCCT  1289
```

>XM_017001128.1:1138-1159 PREDICTED: Homo sapiens BMP/retinoic acid inducible neural specific 3 (BRINP3), transcript variant X5, mRNA

```
Query  8     ACGCTTTACtttttcttttttt  29
             ||||||| ||||| ||||||||
Sbjct  1138  ACGCTTTTCTTTTCCTTTTTTT  1159
```

>NM_145205.5:780-801 Homo sapiens high mobility group box 4 (HMGB4), transcript variant 1, mRNA

```
Query  15   ACtttttctttttttCTCTTCC  36
            |||||||| | |||||||||||
Sbjct  780  ACTTTTTCATCTTTTCTCTTCC  801
```

>NM_153210.4:4144-4180 Homo sapiens ubiquitin specific peptidase 43 (USP43), transcript variant 1, mRNA

```
Query  17    tttttctttttttCTCTTCCTGTGCAGGATTATCTTA  53
             ||||| ||||||| | ||| ||  ||||||| | |||
Sbjct  4180  TTTTTTTTTTTTTTTTTTTCATGGACAGGATTTATTA  4144
```

>NM_005732.3:1252-1278 Homo sapiens RAD50 double strand break repair protein (RAD50), mRNA

```
Query  26    ttttCTCTTCCTGTGCAGGATTATCTT  52
             ||||||||||| || ||  ||||||||
Sbjct  1278  TTTTCTCTTCCAGTTCACTATTATCTT  1252
```

>NM_018111.2:721-755 Homo sapiens chromosome 19 open reading frame 73 (C19orf73), mRNA

```
Query  17   tttttctttttt----tCTCTTCCTGTGCAGGATT  47
            ||||| ||||||    ||||||||||| ||| |||
Sbjct  755  TTTTTTTTTTTTGCACTCTCTTCCTGTACAGTATT  721
```

Ecr, reverse transcription, S (Ser) transcription to C (cytidine); transcription of reversed protein sequence

UUUACUGCCAUAAUGCAUAUCCAUUCUAUUAGGACGUGUCCUUCUCUUUUUUUCUUUUUCAUUUCGCAACUUCAU

>NM_001003681.2:1336-1366 Homo sapiens HMG-box containing 4 (HMGXB4), transcript variant 1, mRNA

```
Query  37    TGTCCTTCTCt--ttttttctttttCATTTC  65
             |||||||||||  ||||||||||||  ||||
Sbjct  1366  TGTCCTTCTCTTCTTTTTTCTTTTTTTTTTC  1336
```

>NM_001136557.1:2319-2340 Homo sapiens G protein-coupled receptor 107 (GPR107), transcript variant 1, mRNA

```
Query  45    TCtttttttctttttCATTTCG  66
             |||||||||||| |||||||||
Sbjct  2319  TCTTTTTTTCTTCTTCATTTCG  2340
```

>NM_001020818.2:2458-2483 Homo sapiens myeloid associated differentiation marker (MYADM), transcript variant 1, mRNA

```
Query  39    TCCTTCTCtttttttctttttCATTT  64
             ||||||| ||||||| |||||| |||
Sbjct  2483  TCCTTCTTTTTTTTTTTTTTTCTTTT  2458
```

>NM_001272063.1:2588-2613 Homo sapiens MAGE family member D4 (MAGED4), transcript variant 1, mRNA

```
Query  47    tttttttctttttCATTTCGCAACTT  72
             ||||||| ||||| |||| |||||||
Sbjct  2613  TTTTTTTTTTTTTAATTTTGCAACTT  2588
```

>NM_030801.3:2588-2613 Homo sapiens MAGE family member D4B (MAGED4B), transcript variant 1, mRNA

```
Query  47    tttttttctttttCATTTCGCAACTT  72
             ||||||| ||||| |||| |||||||
Sbjct  2613  TTTTTTTTTTTTTAATTTTGCAACTT  2588
```

>XM_017010452.2:3849-3871 PREDICTED: Homo sapiens phosphatase and actin regulator 1 (PHACTR1), transcript variant X1, mRNA

```
Query  37    TGTCCTTCTCtttttttcttttt  59
             || |||||||||||||| |||||
Sbjct  3849  TGGCCTTCTCTTTTTTTTTTTTT  3871
```

>NM_032217.4:4564-4591 Homo sapiens ankyrin repeat domain 17 (ANKRD17), transcript variant 1, mRNA.

```
Query  41    CTTCTCtttttttctttttCATTTCGCA  68
             |||||||||| || ||| || |||||||
Sbjct  4591  CTTCTCTTTTCTTTTTTCTCTTTTCGCA  4564
```

>NM_001012754.3:2279-2305 Homo sapiens NHL repeat containing 3 (NHLRC3), transcript variant 1, mRNA

```
Query  39    TCCTTCTC--tttttttctttttCATT  63
             ||||||||  |||||||||||||| ||
Sbjct  2279  TCCTTCTCTTTTTTTTTCTTTTTCTTT  2305
```

>NM_005544.2:8204-8231 Homo sapiens insulin receptor substrate 1 (IRS1), mRNA

```
Query  47    tttttttctttttCATTTCGCAACTTCA  74
             |||||||||||||| |||   |||||||
Sbjct  8204  TTTTTTTCTTTTTCTTTTTAGAACTTCA  8231

>NM_001008701.2:5631-5650 Homo sapiens adhesion G protein-coupled receptor
L1 (ADGRL1), transcript variant 1, mRNA
Query  47    tttttttctttttCATTTCG  66
             |||||||||||| |||||||
Sbjct  5650  TTTTTTTCTTTTCCATTTCG  5631


>NM_001323027.1:217-237 Homo sapiens IQ motif containing G (IQCG),
transcript variant 3, mRNA
Query  39   TCCTTCTCtttttttctttttC  60
            |||||||| |||||||||||||
Sbjct  217  TCCTTCTC-TTTTTTCTTTTTC  237

>NM_001145442.1:6362-6383 Homo sapiens POTE ankyrin domain family member M
(POTEM), mRNA
Query  37    TGTCCTTCTCtttttt-tcttt  57
             |||||||||||||||| |||||
Sbjct  6383  TGTCCTTCTCTTTTTTATCTTT  6362

>NM_018051.4:3765-3784 Homo sapiens WD repeat domain 60 (WDR60), transcript
variant 1, mRNA
Query  47    tttttttctttttCATTTCG  66
             ||||||| ||||||||||||
Sbjct  3784  TTTTTTTTTTTTTCATTTCG  3765

>NM_001081550.1:2795-2818 Homo sapiens THO complex 2 (THOC2), mRNA
Query  39    TCCTTCTCtttttttctttttCATTT  64
             |||||||| |||||||||||| ||||
Sbjct  2818  TCCTTCTC-TTTTTTCTTTTT-ATTT  2795

>XM_017010578.2:970-986 PREDICTED: Homo sapiens zinc finger protein 292
(ZNF292), transcript variant X3, mRNA
Query  40   CCTTCTCtttttttctt  56
            |||||||||||||||||
Sbjct  970  CCTTCTCTTTTTTTCTT  986


>NM_001114132.1:719-746 Homo sapiens neurobeachin like 1 (NBEAL1), mRNA
Query  39   TCCTTCTCttttttt---ctttttCATT  63
            |||||||||||||||   |||||| |||
Sbjct  746  TCCTTCTCTTTTTTTTTGCTTTTTAATT  719

>NM_031892.2:8-34 Homo sapiens SH3 domain containing kinase binding protein
1 (SH3KBP1), transcript variant 1, mRNA
Query  47  tttttttctttttCATTTCGCAACTTC  73
           ||||||| |||||  |||||||| |||
Sbjct  8   TTTTTTTTTTTTTTTTTTCGCAATTTC  34

>NM_052916.2:3004-3025 Homo sapiens ring finger protein 157 (RNF157),
transcript variant 1, mRNA
Query  39    TCCTTCTCtttttttctttttC  60
             ||||||| ||||||| ||||||
Sbjct  3025  TCCTTCTTTTTTTTTTTTTTTC  3004
```

```
>NM_170736.2:44-65 Homo sapiens potassium voltage-gated channel subfamily J
member 15 (KCNJ15), transcript variant 1, mRNA
Query  39  TCCTTCTCttttttttcttttC  60
           ||||||||| ||||||| ||||
Sbjct  44  TCCTTCTCTCTTTTTCTCTTTC  65


>XM_011535661.2:968-994 PREDICTED: Homo sapiens AFG1 like ATPase (AFG1L),
transcript variant X9, mRNA
Query  47   ttttttcttttttCATTTCGCAACTTC  73
            |||||| |||||| ||||  |||||||
Sbjct  968  TTTTTTACTTTTTTATTTTTCAACTTC  994
>NM_020700.1:2197-2213 Homo sapiens protein phosphatase, Mg2+/Mn2+
dependent 1H (PPM1H), mRNA
Query  40    CCTTCTCtttttttctt  56
             |||||||||||||||||
Sbjct  2197  CCTTCTCTTTTTTTCTT  2213

>NM_024577.3:7479-7505 Homo sapiens SH3 domain and tetratricopeptide
repeats 2 (SH3TC2), mRNA
Query  37    TGTCCTTCTCttttttttcttttCATT  63
             ||||||| |||| ||| ||||| ||||
Sbjct  7479  TGTCCTTTTCTTATTTGCTTTTCCATT  7505

>NM_130797.3:36-65 Homo sapiens dipeptidyl peptidase like 6 (DPP6),
transcript variant 1, mRNA
Query  9   CGCTTTA-GttttctttttttCTCTTCCT  37
           ||||||| ||||||  |||||| |||||||
Sbjct  36  CGCTTTATGTTTTTGGTTTTTTTTCTTCCT  65
```

Eg transcription，S (Ser) transcription to G (guanosine)

UAGUUGAACGCUUUAGUUUUUCUUUUUUUCUCUUCCUGUGCAGGAUUAUGUUACGUAUAGGUAAUAGGGUCAUUU

```
>NM_014979.3:10350-10378 Homo sapiens synaptic vesicle glycoprotein 2C
(SV2C), transcript variant 1, mRNA


Query  10     GCTTTAGttttctttttttCTCTTCCTGT  39
              ||||| ||||| |||||||  |||||||||
Sbjct  10350  GCTTT-GTTTTCCTTTTTTATTCTTCCTGT  10378

>NM_144599.4:5836-5859 Homo sapiens NIPA magnesium transporter 1 (NIPA1),
transcript variant 1, mRNA
Query  5     TGAACGCTTTAGttttctttttt  29
             |||| ||||||||||| ||||||||
Sbjct  5836  TGAA-GCTTTAGTTTCCTTTTTTT  5859

>NM_000208.3:7537-7561 Homo sapiens insulin receptor (INSR), transcript
variant 1, mRNA
Query  5     TGAACGCTTTAGttttctttttt  29
             ||||||||||  ||||| |||||||
Sbjct  7537  TGAACGCTTTTTTTTTTTTTTTTTT  7561

>XM_024453260.1:404-428 PREDICTED: Homo sapiens zinc finger protein 648
(ZNF648), transcript variant X2, mRNA
Query  4    TTGAACGCTTTAGttttcttttt  28
            ||||||||| || ||||| ||||||
```

```
Sbjct  404  TTGAACGCTGTATTTTTTTTTTTTT  428

>NM_001318503.1:1961-1985 Homo sapiens dual specificity phosphatase 9
(DUSP9), transcript variant 1, mRNA
Query  12    TTTAGttttctttttttCTCTTCC  36
             ||| |||||| |||||| |||||||
Sbjct  1961  TTTTGTTTTTTTTTTTTCCTCTTCC  1985

>NM_001278209.1:3549-3581 Homo sapiens nucleoporin 153 (NUP153), transcript
variant 1, mRNA
Query  15    AGttttctttttttCTCTTCCTGTGCAGGATTA  48
             ||||  ||||| ||| ||||||||| || |||||
Sbjct  3581  AGTTCCTCTTTCTTT-TCTTCCTGTCCAAGATTA  3549

>XM_024450222.1:2904-2930 PREDICTED: Homo sapiens aspartate beta-
hydroxylase domain containing 1 (ASPHD1), transcript variant X1, mRNA
Query  14    TAGttttctttttttCTCTTCCTGTG  40
             |||| ||| ||| |||| |||||||||
Sbjct  2930  TAGTGTTTTTTTGTTTCCCTTCCTGTG  2904

>XM_017023106.2:1941-1967 PREDICTED: Homo sapiens potassium channel
tetramerization domain containing 13 (KCTD13), transcript variant X8, mRNA
```

Egr transcription, S (Ser) transcription to G (guanosine); transcription of reversed protein sequence

UUUACUGGGAUAAUGGAUAUGCAUUGUAUUAGGACGUGUCCUUCUCUUUUUUUCUUUUUGAUUUCGCAAGUUGAU

```
>NM_001320987.2:4457-4485 Homo sapiens tripartite motif containing 37
(TRIM37), transcript variant 3, mRNA
Query  47    tttttttcttttGATTTCGCAAGTTGAT  75
             ||||||| |||||  |||| |||||||||
Sbjct  4485  TTTTTTTTTTTTTATTTCTCAAGTTGAT  4457

>XM_017007191.1:3587-3612 PREDICTED: Homo sapiens zinc finger protein 385D
(ZNF385D), transcript variant X1, mRNA

Query  39    TCCTTCTCtttttttctttttGATTT  64
             ||||||| ||||||| |||||| |||
Sbjct  3612  TCCTTCTTTTTTTTTGTTTTTGTTTT  3587

>NM_001240.3:6383-6402 Homo sapiens cyclin T1 (CCNT1), transcript variant
a, mRNA
Query  38    GTCCTTCTCtttttttcttt  57
             ||||||||||||||||| ||
Sbjct  6402  GTCCTTCTCTTTTTTTCATT  6383

>NM_016932.4:1509-1535 Homo sapiens SIX homeobox 2 (SIX2), mRNA
Query  39    TCCTTCTCtttttttctttttGATTTC  65
             |||||||||||| || ||| || ||||
Sbjct  1509  TCCTTCTCTTTTCTTTTTTCTGCTTTC  1535

>NM_024734.3:11105-11126 Homo sapiens calmin (CLMN), mRNA
Query  40     CCTTCTCtttttttctttttGA  61
              ||||||||  ||||||||||||
Sbjct  11126  CCTTCTCTACTTTTCTTTTTGA  11105
```

```
>NM_005643.3:1555-1581 Homo sapiens TATA-box binding protein associated
factor 11 (TAF11), transcript variant 1, mRNA
Query  47    tttttttctttttGATTTCGCAAGTTG  73
             ||||||| |||||  ||| ||||||||
Sbjct  1581  TTTTTTTTTTTTTTTTTTTGCAAGTTG  1555

>NM_030628.1:3270-3296 Homo sapiens integrator complex subunit 5 (INTS5),
mRNA
Query  47    tttttttctttttGATTTCGCAAGTTG  73
             ||||||| |||| | |||| |||||||
Sbjct  3296  TTTTTTTTTTTTGGTTTTCTCAAGTTG  3270
```
